# Pulmonary Hypertension in Acute and Chronic High Altitude Maladaptation Disorders

**DOI:** 10.3390/ijerph18041692

**Published:** 2021-02-10

**Authors:** Akylbek Sydykov, Argen Mamazhakypov, Abdirashit Maripov, Djuro Kosanovic, Norbert Weissmann, Hossein Ardeschir Ghofrani, Akpay Sh. Sarybaev, Ralph Theo Schermuly

**Affiliations:** 1 Member of the German Center for Lung Research (DZL), Department of Internal Medicine, Excellence Cluster Cardio-Pulmonary Institute (CPI), Justus Liebig University of Giessen, Aulweg 130, 35392 Giessen, Germany; Akylbek.Sydykov@innere.med.uni-giessen.de (A.S.); Argen.Mamazhakypov@innere.med.uni-giessen.de (A.M.); Norbert.Weissmann@innere.med.uni-giessen.de (N.W.); Ardeschir.Ghofrani@innere.med.uni-giessen.de (H.A.G.); 2 National Center of Cardiology and Internal Medicine, Department of Mountain and Sleep Medicine and Pulmonary Hypertension, Bishkek 720040, Kyrgyzstan; ra.maripov@mail.ru (A.M.); ak_sar777@mail.ru (A.S.S.); 3 Kyrgyz-Indian Mountain Biomedical Research Center, Bishkek 720040, Kyrgyzstan; 4 Department of Pulmonology, Sechenov First Moscow State Medical University (Sechenov University), 119992 Moscow, Russia; djurokos13@gmail.com

**Keywords:** high altitude, hypoxic pulmonary vasoconstriction, high altitude pulmonary edema, pulmonary hypertension, chronic mountain sickness

## Abstract

Alveolar hypoxia is the most prominent feature of high altitude environment with well-known consequences for the cardio-pulmonary system, including development of pulmonary hypertension. Pulmonary hypertension due to an exaggerated hypoxic pulmonary vasoconstriction contributes to high altitude pulmonary edema (HAPE), a life-threatening disorder, occurring at high altitudes in non-acclimatized healthy individuals. Despite a strong physiologic rationale for using vasodilators for prevention and treatment of HAPE, no systematic studies of their efficacy have been conducted to date. Calcium-channel blockers are currently recommended for drug prophylaxis in high-risk individuals with a clear history of recurrent HAPE based on the extensive clinical experience with nifedipine in HAPE prevention in susceptible individuals. Chronic exposure to hypoxia induces pulmonary vascular remodeling and development of pulmonary hypertension, which places an increased pressure load on the right ventricle leading to right heart failure. Further, pulmonary hypertension along with excessive erythrocytosis may complicate chronic mountain sickness, another high altitude maladaptation disorder. Importantly, other causes than hypoxia may potentially underlie and/or contribute to pulmonary hypertension at high altitude, such as chronic heart and lung diseases, thrombotic or embolic diseases. Extensive clinical experience with drugs in patients with pulmonary arterial hypertension suggests their potential for treatment of high altitude pulmonary hypertension. Small studies have demonstrated their efficacy in reducing pulmonary artery pressure in high altitude residents. However, no drugs have been approved to date for the therapy of chronic high altitude pulmonary hypertension. This work provides a literature review on the role of pulmonary hypertension in the pathogenesis of acute and chronic high altitude maladaptation disorders and summarizes current knowledge regarding potential treatment options.

## 1. Introduction

A large portion of human population has inhabited high altitude settings, such as the mountainous geographic locations of the Andes, Tibet, Ethiopian highlands, Pamir, and Tian-Shan. In addition, the number of people traveling to high altitudes in connection with economic or recreational purposes has been constantly increasing during last decades [1]. High altitude is one of the most important extreme environments, characterized by many challenges [2]. Alveolar hypoxia is the most prominent among them, with recognized consequences for the cardio-pulmonary system, including development of pulmonary hypertension [3]. Acute pulmonary hypertension due to an exaggerated hypoxic pulmonary vasoconstriction (HPV) contributes to high altitude pulmonary edema (HAPE), a life-threatening disorder occurring at high altitudes in non-acclimatized healthy individuals [4]. Chronic exposure to high altitude hypoxia induces pulmonary vascular remodeling and development of sustained pulmonary hypertension, which places an increased pressure load on the right ventricle, leading to right heart failure and premature death [5]. This work provides a literature review on the role of pulmonary hypertension in the pathogenesis of acute and chronic high altitude maladaptation disorders and summarizes current knowledge regarding potential treatment options.

## 2. Hypoxic Pulmonary Vasoconstriction

HPV is a unique response of the pulmonary circulation to alveolar hypoxia. Acute HPV is considered as an adaptive response of the pulmonary circulation to a regional alveolar hypoxia, which optimizes ventilation-perfusion matching and gas exchange by diverting blood flow from poorly ventilated to optimally ventilated lung segments [6,7]. Local alveolar hypoxia induces acute HPV, which is limited to the affected lung segments. Importantly, local HPV is not accompanied by an increase in pulmonary artery pressure (PAP). In contrast, during global alveolar hypoxia, which occurs at high altitude, HPV involves the entire pulmonary circulation, resulting in enhanced pulmonary vascular resistance and elevation of PAP.

The exact underlying mechanisms of HPV have not been fully elucidated yet [8]. Nevertheless, it is well recognized that oxygen sensing and signal transduction machinery is located in the arterial smooth muscle cells of the pulmonary precapillary vessels [9]. Accumulating evidence implicates reactive oxygen species generated by mitochondria as essential mediators in HPV [10]. Reactive oxygen species may induce intracellular calcium increase and subsequent contraction of pulmonary artery smooth muscle cells via direct or indirect interactions with other mediators [11].

In isolated buffer-perfused lungs and isolated pulmonary artery rings, hypoxia elicits a biphasic response consisting of a transient vasoconstriction lasting about 10–15 min, followed by a sustained constriction that develops more gradually to reach a plateau after 30–40 min [3,12]. Interestingly, pulmonary vascular response to acute hypoxia in humans has a similar pattern. It consists of two distinct components: a rapid vasoconstriction occurring within a few seconds with a maximal PAP increase at 15 min, followed after about 40 min by a secondary, more gradual PAP elevation, reaching a plateau at 2 h and lasting for at least 8 h [13,14]. A significant variation in the pulmonary vascular response to acute hypoxia has been demonstrated both between and within species [6,15]. In humans, the individual acute HPV also varies significantly [15,16,17].

## 3. Pulmonary Circulation in Sea Level Residents upon Acute Exposure to High Altitude

For most mammals, including humans, the ascent to high altitude is associated with an increase in PAP. Acute HPV is responsible for the initial PAP elevation on exposure to high altitude hypoxia [18]. Breathing 10–12% hypoxic gas mixtures or simulated ascent in a hypobaric chamber leads to a moderate increase in PAP [19,20,21,22]. Ascent to a terrestrial altitude of 3800–4600 m is associated with an elevation of mean PAP up to 20–25 mmHg and systolic PAP up to 30–40 mmHg [23,24,25,26]. However, a more pronounced increase in PAP in response to acute hypoxia is observed in some healthy sea level residents. Interestingly, individuals with a history of HAPE display an exaggerated PAP response to hypoxia [24,27,28].

### 3.1. Factors Modulating the Magnitude of the Hypoxic Pulmonary Vascular Responses

**Figure 1:**
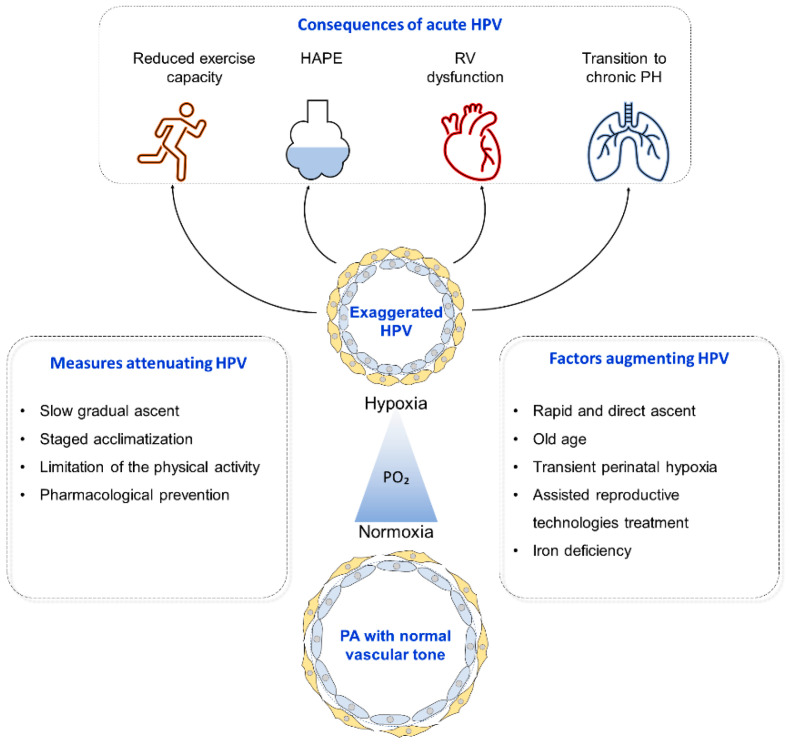
Hypoxic pulmonary vasoconstriction. Hypoxic pulmonary vasoconstriction (HPV) is characterized by constriction of resistive pulmonary arteries (PA) upon exposure to hypoxia. Several factors have been shown to augment HPV, such as rapid and direct ascent to high altitude (HA), old age, transient perinatal hypoxia, assisted reproductive treatment, and iron deficiency. Some measures have been shown to attenuate HPV, such as slow gradual ascent, staged acclimatization, limitation of physical activity, and pharmacological prevention. Exaggerated HPV has been shown to result in several adverse consequences, including reduced physical activity, high altitude pulmonary edema (HAPE) development, right ventricular (RV) dysfunction, and, in the case of prolonged stay at hypoxic condition, HPV may transit to chronic pulmonary hypertension (PH).

Several factors have been identified that modulate the magnitude of the HPV in lowlanders (Figure 1). Children conceived by assisted reproductive technologies [29,30] and young adults who had transient perinatal hypoxic pulmonary hypertension have been shown to display an augmented PAP increase at high altitude [31]. Further, older men exhibit a significantly greater rise in PAP during acute alveolar hypoxia than younger men [32]. A small study showed that females exhibit a greater pulmonary vascular response to sustained hypoxia than males [33]. However, another study did not reveal any significant differences in the pulmonary vascular responses to acute normobaric hypoxia between men and women [34].

There is evidence that continuous or intermittent altitude preexposure can reduce acute mountain sickness and improve physical performance at high altitude [35]. In this regard, ischemic preconditioning of an extremity using an arterial occlusive cuff to one thigh for 5 min, followed by deflation for 5 min for 4 cycles, was associated with blunted PAP elevation in response to hypoxic air breathing [36] and at high altitude [37]. However, these findings were not confirmed by others [38]. Interestingly, staged ascent was associated with a diminished PAP increase at high altitude compared to a direct ascent to the same altitude [39].

Iron bioavailability is another factor that can influence the magnitude of the hypoxic pulmonary vascular pressure response. Iron deficiency can lead to increased pulmonary vascular resistance [40] and potentiate HPV [41,42]. In contrast, elevation of iron stores attenuates pulmonary vascular response to acute hypoxia [41,42,43,44]. Furthermore, supplemental iron attenuated pulmonary hypertension [45] and improved right ventricular function [46] in sea level residents at high altitude.

### 3.2. Pulmonary Hypertension in HIgh Altitude Pulmonary Edema

**Figure 2:**
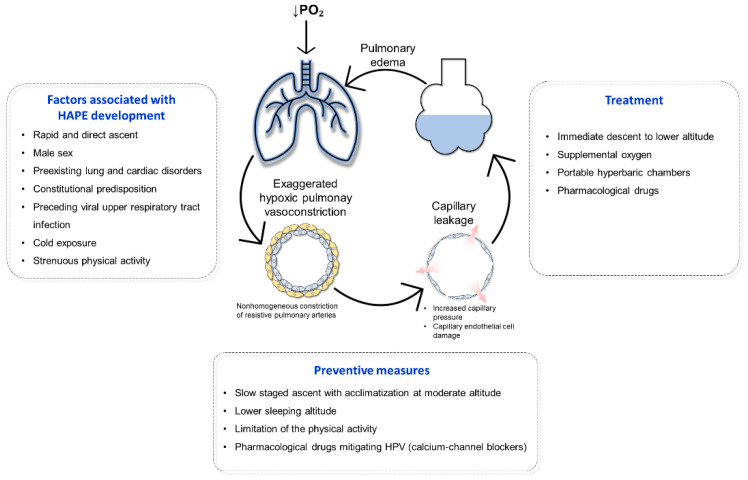
High altitude pulmonary edema. High altitude pulmonary edema (HAPE) develops upon acute exposure to high altitude hypoxia due to exaggerated hypoxic pulmonary vasoconstriction (HPV) and increased capillary damage and leakage due to increased capillary hydrostatic pressure. Several factors have been shown to be associated with increased incidence HAPE, including rapid and direct ascent, male sex, preexisting lung and cardiac disorders, constitutional predisposition, preceding viral upper respiratory tract infection, cold exposure, and strenuous physical activity. Few preventive measures have been shown to attenuate HPV and prevent HAPE development such as slow staged ascent with acclimatization at moderate altitude, lower sleeping altitude, limitation of physical activities, and pharmacological drugs mitigating HPV. Several management strategies have been developed to treat HAPE, such as immediate descent to lower altitude, supplemental oxygen, portable hyperbaric chambers, and pharmacological drugs.

HAPE is a potentially life-threatening form of noncardiogenic pulmonary edema that may develop in otherwise healthy individuals during the first days after rapid ascent to high altitudes [47]. Risk factors for HAPE include the rate of ascent, male sex, and preexisting lung or cardiac disorders (Figure 2) [27,48,49]. The high recurrence rate of HAPE in some individuals suggests a constitutional predisposition [50,51]. Interestingly, most HAPE-susceptible individuals are characterized by an abnormal rise in PAP in response to acute hypoxia [52,53,54,55,56]. Longer duration of the acute hypoxic exposure (2 h vs. 15 min) at low altitude is associated with less overlap between HAPE-susceptible and HAPE-resistant subjects and allows a better discrimination between them [27]. Doppler echocardiography studies showed that about 10% of a healthy Caucasian population display an exaggerated HPV that is comparable in magnitude to the response measured in HAPE-susceptible individuals [57]. These figures are similar to the prevalence of HAPE in the general population when climbing to altitudes of 4000–5000 m in a single day [4].

Exaggerated hypoxic pulmonary hypertension is a hallmark of HAPE. Early studies with invasive hemodynamic measurements documented the presence of an abnormally high PAP in HAPE patients [58,59,60]. Further, numerous clinical observations revealed a relationship between the occurrence of HAPE and the degree of PAP elevation [61,62], suggesting an essential role of pulmonary hypertension in the pathogenesis of this condition. This view is further supported by the reduced HAPE incidence in susceptible subjects [63,64] and clinical improvement in HAPE patients by drugs lowering PAP [64,65,66] or treatment in hypobaric chambers [67,68]; development of HAPE at lower altitudes in patients with various anomalies and diseases, predisposing them to pulmonary hypertension [69,70,71,72]; individual susceptibility to HAPE, which is associated with an exaggerated pulmonary vascular response to hypoxia [53,55] and during exercise in normoxia [28,54].

In experimental studies, the nonhomogeneous nature of HPV has been demonstrated [73,74]. Furthermore, HAPE-susceptible subjects display uneven distribution of pulmonary blood flow in response to acute hypoxia [75,76,77]. Nonhomogeneous HPV in HAPE leads to reduced blood flow in lung regions, with stronger vasoconstriction and overperfusion in those with weaker pulmonary vasoconstriction [62,78]. This idea is supported by clinical observations of focal pulmonary edema in areas with intact pulmonary arterial blood flow in patients with preserved cardiac function after massive acute pulmonary embolism [79]. Increased flow through the less constricted vessels due to nonhomogeneous pulmonary vasoconstriction can lead to elevation of the capillary pressure and hydrostatic lung edema. In a prospective study, a rise in microvascular pressure and non-impaired pulmonary capillary permeability during first days of high altitude acclimatization was demonstrated in HAPE-susceptible subjects [24]. Increased capillary pressure can cause disruption of the capillary endothelium, alveolar epithelium, or sometimes all layers, leading to leaking of high molecular weight proteins and erythrocytes into the interstitial and alveolar spaces [80,81,82,83]. High pressure-induced damage of pulmonary capillaries could explain the protein-rich nature of the lavage fluid in HAPE patients [84]. Importantly, this damage is reversible, and the integrity of the capillary membrane can be restored within minutes following a reduction in pressure [85].

Although most of the HAPE-susceptible subjects exhibit abnormal pulmonary vascular responses to hypoxia [27,86], some studies have suggested that an exaggerated pulmonary hypertension is not sufficient to trigger HAPE [87,88]. In line with this notion, patients with idiopathic pulmonary arterial hypertension do not develop pulmonary edema. However, development of HAPE in persons with an exaggerated HPV can be provoked by the concurrent presence of several other HAPE risk factors, such as rapid ascent, the absolute altitude difference gained, pre-acclimatization, preceding viral upper respiratory tract infection, exposure to cold, and engagement in strenuous physical activity during the first days of acclimatization to high altitude [48,62,89,90,91]. Earlier observations reported more frequent occurrence of HAPE in men than in women [62,92]. But no significant differences were revealed in the pulmonary vascular responses to acute normobaric hypoxia between men and women [34].

### 3.3. Prevention and Treatment of Pulmonary Hypertension in High Altitude Pulmonary Edema

#### 3.3.1. Non-Pharmacological Prevention of Pulmonary Hypertension in High Altitude Pulmonary Edema

The most efficient approach to prevent or reduce HAPE severity is a properly organized acclimatization process. Most important is the sleeping altitude and not the highest elevation achieved during the day [93]. It is recommended to reduce physical activity during the first days of acclimatization. Intense physical exercise can provoke HAPE [89] by PAP elevation due to increased cardiac output. Further, rigorous physical activity can be associated with stress capillary failure in humans and some animals [94,95]. Although pulmonary edema develops rarely at sea level in humans [96], intense physical activity in hypoxia can lead to stress capillary failure and pulmonary edema [97].

For successful acclimatization, a slow staged ascent is recommended with the first sleep at an altitude below 2400 m. Prospective studies demonstrated reduced incidence and severity of acute mountain sickness during slow staged ascent [98,99]. Furthermore, staged ascent with an acclimatization at moderate altitude is associated with an attenuated PAP increase at high altitude compared to that associated with the direct ascent to high altitude [39]. Indeed, gradual ascent to high altitude can prevent HAPE in most HAPE-susceptible persons [48]. Thus, individuals with an exaggerated PAP elevation in response to acute hypoxia can successfully acclimatize to high altitude by controlling the rate of ascent and avoiding unprotected exposure to extreme environmental cold and strenuous exercise during the first days at altitude. However, in emergency cases during rescue and military operations, a rapid ascent may be required, which can compromise successful high altitude acclimatization [100]. In this situation, prevention of HAPE by pharmacological means might be necessary.

#### 3.3.2. Pharmacological Prevention of Pulmonary Hypertension in High Altitude Pulmonary Edema

One of the key biologically active mediators regulating vascular tone is nitric oxide. Nitric oxide is produced by the enzyme nitric oxide synthase, located in the endothelial vascular cells and epithelial cells of the airways. Local generation of nitric oxide (NO) contributes to blood distribution from poorly ventilated regions to well aerated ones, thus maintaining normal ventilation-perfusion matching. There is experimental evidence of hypoxia-induced suppression of NO synthase activity [101,102,103]. Inhibition of the NO synthase potentiates HPV [104], whereas enhancing NO bioavailability by its inhalation [104,105] or phosphodiestherase-5 inhibition [20] attenuates HPV. Furthermore, mice with genetic deficiency of endothelial NO synthase develop more prominent acute hypoxic pulmonary hypertension compared to wild-type mice [106]. All these suggest that impaired NO synthesis and/or bioavailability might underlie exaggerated HPV in HAPE-susceptible subjects.

In HAPE-susceptible subjects, acute hypoxia leads to decreased exhaled NO levels [107], and high altitude sojourn is associated with a greater reduction in exhaled NO levels compared to HAPE-resistant individuals [108]. There is also an inverse correlation between PAP and exhaled NO levels [107,108]. During the first days at high altitude, there is a decrease of the transpulmonary gradient of NO metabolites [109], suggesting reduced production or increased inactivation of NO in the lungs. Further, plasma levels of L-citrulline, a marker of NO production, are reduced at high altitude, and intravenous infusion of L-arginine, a substrate for endothelial NO synthase, results in increased L-citrulline plasma levels and improved gas exchange in healthy individuals at high altitude [110]. Another study demonstrated that the severity of high altitude pulmonary hypertension is associated with reduced NO bioavailability due to increased reactive oxygen species in the lungs [111]. In line with these observations, increasing NO bioavailability by its inhalation led to significant reduction in PAP in HAPE patients [112,113].

Several randomized double-blind placebo-controlled cross-over studies clearly demonstrated attenuation of hypoxic PAP elevation in healthy volunteers at rest and during exercise by a single dose of the phosphodiestherase-5 inhibitor sildenafil [20,114,115,116]. Moreover, in randomized double-blind placebo-controlled studies, sildenafil reduced systolic PAP at rest and during exercise in healthy individuals at high altitude [114,116,117].

Endothelin-1 is one of the most potent vasoconstrictor agents [118]. Endothelin-1 plays an important role in the regulation of pulmonary blood flow, and its enhanced production and/or decreased clearance might contribute to pulmonary hypertension. Hypoxia, ischemia, and acute stress are the main stimulators of endothelin-1 production [119]. High altitude ascent is associated with a rise in plasma levels of endothelin-1, which is correlated with the magnitude of PAP elevation [120,121]. At high altitude, there was a transpulmonary increase of plasma endothelin-1 in mountaineers, suggesting enhanced generation and/or decreased clearance of endothelin-1 in the hypoxic lungs [109]. Furthermore, a correlation between pulmonary hypertension severity and endothelin-1 levels has been demonstrated in HAPE-susceptible subjects [122,123,124].

Endothelin-1 binds two types of receptors, A and B. The former is located on endothelial cells; the latter is expressed by endothelial cells and smooth muscle cells. Activation of both receptors on smooth muscle cells induces vasoconstrictor and mitogenic effects of endothelin-1, whereas stimulation of the receptors on endothelial cells contributes to endothelin-1 clearance and nitric oxide and prostacyclin release. Randomized double-blind placebo-controlled cross-over studies demonstrated that a single dose of 250 mg bosentan, a dual endothelin receptor antagonist, significantly attenuated hypoxia-induced PAP rise in healthy volunteers [125] and in individuals with HAPE in the past [126], without negatively affecting blood gases, systemic pressure, and cardiac output. Bosentan in a dose of 130 mg/day not only attenuated hypoxic pulmonary vascular resistance increase but also increased aerobic physical capacity in healthy volunteers [127]. In a randomized blind placebo-controlled study, bosentan in a dose of 250 mg/day reduced PAP during the first days at high altitude [122]. In contrast, no positive effect of bosentan on systolic PAP and physical capacity were revealed in healthy volunteers during exposure to an altitude of 3800 m [128]. Further, randomized double-blind placebo-controlled studies demonstrated that, in healthy sea level residents, the selective endothelin receptor type A antagonist sitaxentan attenuated acute hypoxic pulmonary vascular resistance elevation and reduced pulmonary vascular resistance at high altitude [129]. However, sitaxentan has been withdrawn from the market due to hepatotoxicity. Thus, a number of randomized controlled studies demonstrated usefulness of endothelin receptor antagonists in reducing PAP in healthy sea level residents during high altitude exposure.

Several other drugs have been demonstrated to reduce the elevated PAP at high altitude. In a randomized double-blind placebo-controlled study, nifedipine has been shown to reduce PAP and prevent HAPE in HAPE-susceptible mountaineers [63]. In another randomized double-blind placebo-controlled study, the phosphodiestherase-5 inhibitor tadalafil and dexamethasone both reduced PAP increase and prevented HAPE in subjects with HAPE in the past [64]. The beneficial effects of dexamethasone might be due its capability to increase expression and activity of nitric oxide synthase [130]. In addition, dexamethasone decreases fluid transport across vessel walls [131] and increases alveolar fluid clearance by enhancing expression of epithelial sodium channels and activity of the sodium–potassium ATPase [132,133].

Acetazolamide, a carbonic anhydrase inhibitor, has long been successfully used for the prevention and treatment of acute mountain sickness [134]. The underlying mechanism is not fully understood and may be multifactorial, but it is most likely related to the increased ventilatory drive and improved oxygenation due to metabolic acidosis induced by inhibition of renal carbonic anhydrase [135]. Experimental studies demonstrated that acetazolamide attenuated hypoxia-induced PAP increase [136]. Interestingly, inhibition of HPV by acetazolamide is not related to the inhibition of carbonic anhydrase activity [137]. In a prospective randomized double-blind placebo-controlled crossover study, acetazolamide attenuated PAP increase in response to acute hypoxia in healthy individuals [136].

Thus, a potential role in PAP reduction and HAPE prevention has been demonstrated for a number of drugs targeting various signaling pathways. However, only calcium-channel blockers are currently recommended for drug prophylaxis in high-risk individuals with a clear history of recurrent HAPE due to extensive clinical experience with nifedipine in HAPE prevention in susceptible individuals [138]. Further controlled studies are necessary to evaluate the efficacy of these drugs in HAPE treatment in susceptible individuals.

**Figure 3:**
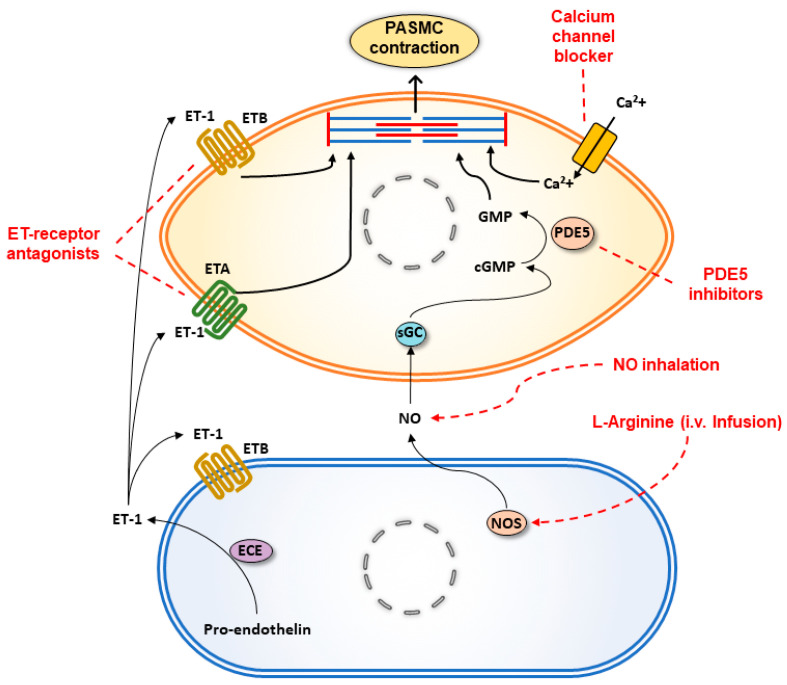
Targets of potential pharmacological agents to prevent or reverse HPV. Upon exposure to hypoxic condition, in pulmonary artery endothelial cells (PAECs), endothelin converting enzyme (ECE) increases endothelin-1 (ET-1) levels, which in turn causes pulmonary artery smooth muscle cell (PASMC) contraction via ET-1 A and B receptors (ETA and ETB). ETA and ETB can be blocked by endothelin receptor antagonists such as bosentan to inhibit hypoxia induced PASMC contraction. Similarly, nitric oxide synthase (NOS) activity is also impaired in hypoxic PAECs, leading to decreased levels of nitric oxide (NO). Further, decreased bioavailability of NO for the activity of soluble guanylate cyclase (sGC) results in decreased levels of cyclic guanosine monophosphate (cGMP) in PASMCs and PASMC contraction. NO level can be restored by either infusion of L-arginine, which is used by NOS as a substrate, or by NO inhalation, which directly activates sGC. To prevent cGMP degradation in PASMCs, phosphodiesterase type 5 (PDE5) can be inhibited by PDE5 inhibitors such as sildenafil and tadalafil. In addition, calcium influx and PASMC contraction can be inhibited with calcium channel blockers such as nifedipine.

Targets of potential pharmacological agents to prevent or reverse HPV are summarized in Figure 3. Summaries of relevant studies evaluating effects of potential drugs on HPV in in vitro, ex vivo, and in vivo rodent models and healthy human subjects exposed to hypoxia are presented in Table 1 and Table 2.

### 3.4. Treatment of Pulmonary Hypertension in High Altitude Pulmonary Edema

Any delay in treatment initiation can lead to fatal consequences. In mild cases, bed rest and supplemental oxygen might be sufficient [155,156]. However, in severe cases, prompt descent is the only effective treatment option [93]. Even descent of 100 m can result in significant patient improvement. However, it is recommended that individuals should try to descend at least 1000 m or until symptoms resolve [157]. When available, before descent, supplemental oxygen is started or the patient is placed in a hyperbaric chamber [68]. Supplemental oxygen is indicated in all cases of HAPE, independent of disease severity [156]. It reduces hypoxia and decreases PAP [93]. If descent is impossible and oxygen is not available, the patient may be placed into hyperbaric chamber [158]. The pressure in the hyperbaric chamber simulates a descent of about 1500 to 2500 m. Improvement is achieved in 2–4 h [159].

Given the important role of HPV and pulmonary hypertension in HAPE pathogenesis, it seems rational to use vasodilators for HAPE treatment. Nitric oxide is one of the potent endogenous vasodilators, which is synthesized by endothelial nitric oxide synthase. Local production of nitric oxide contributes to the maintenance of the normal ventilation-perfusion matching by redistributing blood to the better ventilated lung areas. Indeed, nitric oxide inhalation has been demonstrated to selectively inhibit acute HPV in healthy humans without causing systemic hypotension [160]. Moreover, nitric oxide inhalation significantly reduced PAP and improved radiological signs of pulmonary edema without compromising systemic hemodynamics in HAPE patients [112,113]. However, limited availability of nitric oxide at high altitude is a drawback of this therapy. Although controlled studies supporting the use of phosphodiesterase-5 inhibitors for treatment of HAPE are lacking, their use in the field is relatively common [161,162,163].

The calcium channel antagonist nifedipine has been demonstrated to improve symptoms and radiological evidence of alveolar edema in HAPE patients, which were associated with improved arterial oxygenation and reduction of the alveolar-arterial gradient and PAP in a nonrandomized, unblinded study [66]. In contrast, treatment with nifedipine did not show any advantages compared to placebo in a study of 110 Indian soldiers with HAPE [164]. Late initiation of treatment with nifedipine after admission to the hospital located at low altitude may account for the discrepancy [164]. Despite limited evidence supporting its utility for the treatment of HAPE, nifedipine has been used extensively in the field [138,163].

Although there is no clear recommendation for vasodilator therapy, in field settings, nifedipine should be used as an adjunct to descent, supplemental oxygen, or portable hyperbaric therapy or as primary therapy if none of these other measures are available [157]. If nifedipine is also unavailable, a phosphodiesterase inhibitor can be used [157]. In addition, further evaluation of the efficacy of other potential drugs—such as endothelin receptor antagonists, rho-kinase inhibitors, guanylate cyclase stimulators—as additive or alternative agents for HAPE treatment is warranted [165].

## 4. Pulmonary Circulation in Sea Level Residents Chronically Exposed to High Altitude

Prolonged exposure to alveolar hypoxia leads to remodeling of pulmonary vessels, resulting in sustained pulmonary hypertension. However, most of the studies investigated relatively short periods of high altitude acclimatization in the range of several days to weeks. In Operation Everest II, simulated ascent in a hypobaric chamber of sea level residents for several weeks resulted in a stable rise of PAP, which was partially responsive to oxygen [166]. Likewise, changes in PAP suggestive of pulmonary vascular remodeling were demonstrated in healthy sea level residents following progressive exposure to high altitude during a trek to Everest base camp [167]. Of note, these studies were designed to expose subjects to progressively increasing levels of hypoxia without achieving a steady state at a given altitude.

Invasive PAP measurements have demonstrated that prolonged altitude exposure during a four-week sojourn at 3454 m leads to a persistent elevation in PAP in healthy sea level residents [168]. In most acclimatized lowlanders, increase in PAP is mild-to-moderate [23,169]. Interestingly, comparison of echocardiography-derived mean PAP in healthy Chinese male volunteers demonstrated lower values in those exposed to 3700 m for one year compared to those exposed to the same altitude for 24 h [170]. Furthermore, the proportion of subjects with confirmed pulmonary hypertension was significantly lower in the chronically exposed group compared to the group acutely exposed to high altitude hypoxia [170]. Though this study did not investigate the time course of the PAP changes of individual subjects, its findings suggest that PAP moderately reduces following long-term acclimatization. Indeed, lowlanders have higher PAP values compared to Himalayan high altitude dwellers upon acute exposure to high altitude [171]. However, PAP values in sea level residents were not different from those in Sherpas following three weeks of high altitude acclimatization [172]. Likewise, similar PAP values were revealed in Bolivian high altitude natives and well-adapted Caucasian low altitude natives permanently living at the same high altitude location [173].

In some temporary high altitude residents, clinically significant pulmonary hypertension may develop. Pulmonary hypertension may persist following the resolution of HAPE [174]. However, even severe pulmonary hypertension may remain asymptomatic for longer periods until the patients go to considerably higher altitudes or perform intensive exercises [174]. In sea level residents, the symptoms of pulmonary hypertension develop after staying for 5–42 months at high altitude [174]. In most patients, symptoms of pulmonary hypertension resolve within 1–21 months of return to sea level [174]. Occasionally, the disease may further progress, despite moving to low altitude, and lead to death [174].

About three decades ago, a syndrome characterized by severe pulmonary hypertension and right heart failure was initially described in infants of Han origin who were born in lowlands and subsequently brought to live at altitudes above 3000 m [175]. In the largest reported series of cases, the average age of affected infants was 9 months and the average duration of their stay at high altitude was only 2.1 months [175]. Nowadays, Han mothers residing at high altitude descend to lowland regions to give birth and do not bring their children to high altitude until they have reached more than one year of age [176]. There is now evidence that not only Han Chinese infants are susceptible to this condition but that it can also develop in infants of other origin including Tibetan and Kyrgyz [176,177]. In Tibetans, this condition occurs in infants born to dwellers who migrated from an intermediate altitude to a significantly higher altitude [176]. Comprehensive studies with invasive measurements of pulmonary hemodynamics and postmortem pathological investigations demonstrated sustained pulmonary hypertension and substantial remodeling of small pulmonary arteries as well as hypertrophy and dilatation of the right ventricle in these patients [175,176,178]. This syndrome was coined subacute infantile mountain sickness [179].

In 10–20% of acclimatized sea level residents, prolonged exposure (>3 months) to extreme altitudes of 5800–6700 m is associated with development of subacute adult mountain sickness [179,180]. This syndrome is characterized by severe hypoxic pulmonary hypertension with right ventricular failure [181,182]. Importantly, pulmonary hypertension and signs of right ventricular failure in patients with subacute adult mountain sickness gradually reverse following staying for several weeks at low altitude [181].

## 5. Pulmonary Circulation in Healthy High Altitude Residents

Early studies revealed PAP elevation in permanent high altitude dwellers [183,184,185]. Histological studies revealed structural remodeling of pulmonary vessels in permanent high altitude natives, which were characterized by increased muscularization of distal arteries with extension of smooth muscle cells into previously non-muscularized arterioles [186]. In addition to structural vascular remodeling, persistent vasoconstriction is an important contributor to chronic hypoxia-induced pulmonary hypertension [187]. Interestingly, the relative contribution of persistent vasoconstriction and structural changes in the vasculature to chronic hypoxia-induced pulmonary hypertension varies in different experimental animals [188,189,190]. Moreover, the relative contribution of vasoconstrictor and structural mechanisms to chronic hypoxic pulmonary hypertension may differ between individuals within the same species. Thus, after several months spent at high altitude, administration of oxygen to a steer with moderate pulmonary hypertension reduced PAP to near normal values, whereas in a steer with severe pulmonary hypertension led to only a partial reduction of PAP [191].

Invasive pulmonary hemodynamic measurements in a small number of native Andeans revealed a very close correlation between PAP values and the altitude level of birthplace [192]. This dependence is not linear and applicable to many other high altitude populations [5]. PAP levels remain unchanged below 2000 m [7] and slightly increase up to 3000 m [17,193,194,195]. Above 3000 m, PAP increases more markedly with altitude [192]. A recent systematic review and meta-analysis of studies reporting echocardiographic estimations of PAP in healthy individuals of the general high altitude population, which included 12 studies comprising 834 participants, revealed that for altitudes between 3600 and 4300 m, the mean right ventricular-to-atrial pressure gradient as an estimate of systolic PAP was 25 mmHg, which was about 7 mmHg higher than that of lowland populations [196].

Although the average PAP levels in the high altitude populations are higher than those in sea level residents, there is marked variability in PAP values among highlanders [17,185,197,198], suggesting that not all individuals are prone to PAP elevation at high altitude. Thus, while most highlanders display mild-to-moderate PAP elevation, some high altitude dwellers may have PAP values that are not different from those in sea level residents, and others may develop severe pulmonary hypertension. Likewise, structural changes in the pulmonary vasculature vary significantly between individuals and between different populations [199,200,201]. Moreover, pulmonary vessels of some highlanders do not display changes typical for hypoxia-induced remodeling [202,203]. Similar findings were reported in well-adapted to life at altitude animals including yaks [204,205,206] and lama [207,208], suggesting that very long high altitude residence of the population leads to successful adaptation, and minimal PAP elevation represents one of its features. Indeed, it is currently believed that there are geographical and/or ethnical differences in PAP levels among high altitude populations. For example, earlier studies demonstrated that Tibetans residing at 3658 m display mean PAP of 15 mmHg, which is not different from that in sea level residents [209]. Likewise, mean PAP values of 20 mmHg have been reported for native Ladakh residents of Tibetan origin at 3600 m [210]. Furthermore, small pulmonary arteries of native Himalayan highlanders are thin-walled with no medial hypertrophy of the pulmonary arteries [201]. It should be noted, however, that these studies were performed on very few subjects.

Interestingly, large studies with non-invasive estimation of PAP did not reveal any difference in systolic PAP levels between Han Chinese and Tibetan children aged 0–14 years permanently residing at 3700 m [211]. Similarly, more recent studies did not confirm earlier findings of significant differences in PAP levels among healthy highlanders of various ethnicity residing in different geographical regions [173,212,213,214]. Moreover, no differences in Doppler echocardiography-derived systolic PAP were observed between acclimatized Europeans and Bolivian Aymara or Sherpas [172,173]. Finally, a recent meta-analysis did not support the current belief of differences in PAP levels between different high altitude populations [196]. Remarkably, the meta-analyzed data on arterial oxygen saturation confirmed the previous observation of lower arterial oxygen saturation in Tibetans compared to other highlanders [196]. However, despite lower arterial oxygen saturation, Tibetans do not exhibit higher PAP levels, which may be due to blunted pulmonary vascular responses to both acute and sustained hypoxia in Tibetans [209,215]. One of the underlying mechanisms could be enhanced nitric oxide production in Tibetans [212,216,217], which may increase oxygen delivery, thus compensating for low oxygen content in the blood.

## 6. High Altitude Pulmonary Hypertension and Chronic Mountain Sickness

**Figure 4:**
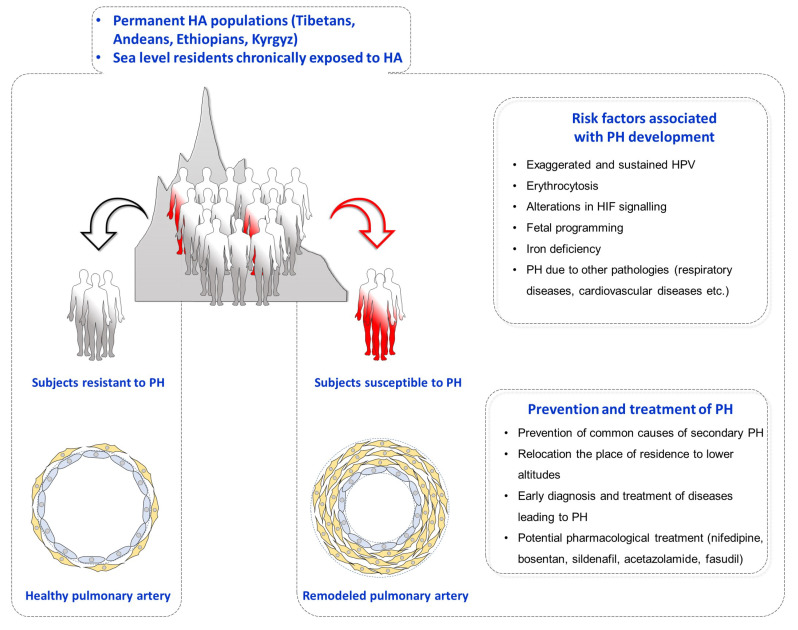
Pulmonary hypertension in high altitude residents. Populations permanently living at high altitude (HA)—such as Tibetans, Andeans, Ethiopians, and Kyrgyz—and sea level residents chronically exposed to HA are at risk for the development of pulmonary hypertension (PH). In addition to chronic alveolar hypoxia, susceptibility to develop PH at HA can be increased by several factors, such as exaggerated and sustained hypoxic pulmonary vasoconstriction (HPV), erythrocytosis, altered HIF signaling, fetal programming, iron deficiency, and due to other respiratory and cardiovascular diseases. Several strategies have been suggested to prevent and treat PH at HA, including prevention of common causes of secondary PH, relocation the place of residence to lower altitude, early diagnosis and treatment of diseases leading in PH, and application of potential pharmacological treatment (nifedipine, bosentan, sildenafil, acetazolamide, and fasudil).

Although elevation of PAP in most high altitude dwellers is modest, some individuals may develop severe pulmonary hypertension [218]. Pulmonary hypertension places an increased pressure load on the right ventricle, leading to right heart failure and premature death. Multiple factors can promote development of pulmonary hypertension in high altitude dwellers, including magnitude of individual HPV, blood viscosity, low ambient temperatures, co-morbidities, geographical location and landscape of the place of residence, seasonal migrations, and others (Figure 4). Epidemiologic observations in the Andes demonstrated that chronic mountain sickness, a high altitude maladaptation disorder, affects mostly males and is rare in females [219,220]. Furthermore, there is a sharp increase in chronic mountain sickness incidence in women after menopause [221]. Likewise, a recent study revealed higher prevalence of high altitude pulmonary hypertension in men than in women [222].

### 6.1. Hypoxia Inducible Factors (HIFs)–Prolyl Hydroxylase Domain (PHD) Enzymes–von Hippel–Lindau Tumor Suppressor (VHL) Protein System

Hypoxia inducible factors (HIFs) play an essential role in oxygen homeostasis by facilitating oxygen supply to the tissues under hypoxic conditions. HIFs coordinate intracellular signaling in response to oxygen deficiency by regulating transcription of hundreds of hypoxia-controlled genes [223]. HIFs are heterodimeric transcription factors comprising a constitutively expressed β subunit (HIF1β) and one of the three oxygen-sensitive subunits: HIF1α, HIF2α, or HIF3α. Under normoxic condition, HIFs are instantaneously inactivated by hydroxylation of proline amino-acid residues within the α subunit leading to polyubiquitination and subsequent proteasomal degradation [224]. HIF prolyl hydroxylation is mediated by the activity of prolyl hydroxylase domain (PHD) enzymes, which generate a binding site for von Hippel–Lindau tumor suppressor (VHL) protein. VHL protein binds to the hydroxylated HIFα and serves as a recognition component of an E3–ubiquitin ligase complex. Thus, PHD proteins and VHL protein are negative regulators of HIFs, and loss-of-function mutations in the gene encoding them impairs ubiquitination and degradation of HIFα, leading to HIFα accumulation [225]. Under hypoxia, the activity of the PHD enzymes is decreased, resulting in stabilization and accumulation of HIFα isoforms, which then build heterodimers with the constitutively expressed HIF1β. This HIF complex binds to hypoxia response elements in DNA, initiating or enhancing transcription of HIF-dependent genes.

At sea level, individuals with various mutations impairing HIF signaling display a phenotype similar to that seen in lowlanders acclimatized to high altitude hypoxia. Activating HIF2α gene mutations are associated with elevated resting PAP values [226,227] and an augmented PAP elevation in response to acute hypoxia [227]. In line with these reports, mildly elevated baseline PAP values and an exaggerated hypoxic pulmonary vascular response was demonstrated in an individual with a heterozygous mutation in PHD2 [228]. Furthermore, a homozygous missense mutation R200W in the VHL gene causes familial erythrocytosis or Chuvash polycythemia, an autosomal recessive disorder endemic to the Russian region of Chuvashia [229,230,231] and on the island of Ischia in Italy [232]. Patients with Chuvash polycythemia were found to exhibit various vascular abnormalities, including increased PAP levels [233,234]. Notably, the elevation in PAP values persisted even after adjustment for the increased blood volume in affected persons [235]. Further, lower ferritin concentration due to phlebotomy independently predicted higher PAP levels in these patients [235]. Recently, some other sporadic missense mutations in the VHL gene have been reported to be associated with erythrocytosis and various degrees of pulmonary hypertension [236,237,238,239]. Furthermore, exposure to acute hypoxia provoked an exaggerated PAP elevation in patients with Chuvash polycythemia and with other VHL mutations [240,241,242].

There is compelling evidence from experimental models implicating the HIF pathway in hypoxic pulmonary hypertension [243,244]. Importantly, pulmonary circulation abnormalities associated with the impaired HIF signaling have been recapitulated in genetically modified mice. Chronic hypoxia-induced pulmonary hypertension was significantly attenuated or delayed in mice hemizygous for either HIF1α [245] or HIF2α [246,247] and in mice with vascular smooth muscle HIF1α [248], endothelial, or global partial HIF2α deletion [249,250,251]. Interestingly, mice with endothelial or global HIF1α or vascular smooth muscle HIF2α deletion were not protected from chronic hypoxic pulmonary hypertension [249,250,251]. The critical role of HIF2α is further supported by the demonstration of spontaneously developed severe pulmonary hypertension in mice with a global HIF2α gain-of-function mutation or with deletion of PHD2 in endothelial cells pulmonary vascular disease [227,247,250,252,253,254]. Moreover, concomitant genetic disruption of endothelial PHD2 and HIF2α in this model completely protected from pulmonary hypertension development [247,253]. In line with these findings, heterozygous deletion of HIF2α, but not HIF1α, suppressed both polycythemia and pulmonary hypertension in the murine model of Chuvash polycythaemia [255]. Further evidence supporting the critical role of HIF2α in the development of hypoxic pulmonary hypertension was provided by demonstration of high association of two EPAS1 variants, which are likely a gain-of-function mutation, with pulmonary hypertension in cattle residing at high altitude [256].

Several genome-wide selection studies provided evidence of natural selection for gene variants encoding proteins in the HIF signaling pathway, EPAS1 (encodes HIF2a), and EGNL1 (encodes PHD2) in high altitude populations [257,258]. Identified genetic polymorphisms in EPAS1 and EGLN1 in Tibetans correlate with hemoglobin concentration. Of note, lower hemoglobin concentration and a lower red cell mass reduces blood viscosity and by this can contribute to a better adaptation of the pulmonary circulation to life at high altitude [3]. In addition, an association between two EPAS1 variants and lower PAP values was recently reported in Tibetans living at high altitude [259].

### 6.2. Variability in Hypoxic Pulmonary Vasoconstriction

The severity of pulmonary hypertension at high altitude is characterized by an interindividual variability, which may result from differences in hypoxic stimulus intensity and/or intrinsic strength of the pulmonary vascular response. The interspecies and intraspecies variability of acute HPV is well recognized [6,7,15]. In cattle, severity of chronic hypoxia-induced PH has been shown to correlate with the strength of acute HPV [260], suggesting that the mechanisms underlying the pulmonary vascular responses during chronic hypoxia are the same or related to those in acute hypoxia. On the contrary, no correlation between the magnitude of acute HPV and the severity of chronic hypoxia-induced pulmonary hypertension was revealed in other species. For example, despite having a vigorous acute HPV [261], coatis do not develop pulmonary hypertension in response to chronic hypoxic exposure [262]. Further, guinea pigs develop moderate chronic hypoxic pulmonary hypertension despite a relatively weak acute HPV [263]. In line with these findings, discordant pulmonary vascular responses to acute and chronic hypoxia were demonstrated in different rat strains [264,265,266]. Finally, recent studies clearly demonstrated that pulmonary vascular responses to acute and chronic hypoxia might be regulated by different signaling pathways [267,268,269]. For example, TRPC6-deficient mice developed chronic hypoxia-induced pulmonary hypertension despite disrupted acute HPV [268]. In contrast, TRPC1 gene deletion did not impair acute HPV while diminishing development of pulmonary hypertension in chronic hypoxia [267].

### 6.3. Erythrocytosis

Exposure to high altitude hypoxia stimulates erythrocytes production in an attempt to improve tissue oxygenation. Long-term high altitude residence can be complicated in some individuals by development of excessive erythrocytosis and chronic mountain sickness [270]. Erythrocytosis elevates resistance to pulmonary blood flow by increasing blood viscosity [271,272,273]. Indeed, perfusion of isolated rat lungs following three weeks of hypoxic exposure with blood with high hematocrit led to a significant elevation of pulmonary vascular resistance [274]. Further, isovolumic hemodilution in highlanders with erythrocytosis was associated with a significant reduction of pulmonary vascular resistance [275,276]. Interestingly, calculations of PAP values in high altitude dwellers, taking into account the hematocrit, revealed that higher values in Andeans are due to higher hematocrit levels compared to Tibetans [277]. In contrast to early studies, a recent meta-analysis revealed that resting PAP in patients with chronic mountain sickness is only slightly higher compared to apparently healthy high altitude dwellers [278]. Nevertheless, in chronic mountain sickness patients, PAP is significantly more accentuated during even mild exercise associated with daily activity [278,279].

### 6.4. Fetal Programming

Several studies have demonstrated that adverse oxygenation during perinatal and late fetal life is associated with higher PAP levels in young, high altitude residents [280,281]. The underlying mechanisms are unclear, but experimental data suggest that epigenetic fetal programming of pulmonary vascular dysfunction might play a role [282].

### 6.5. Iron Deficiency

Several studies demonstrated that manipulation of iron bioavailability can modulate responses of the pulmonary circulation to acute hypoxia [40,41,43]. Interestingly, supplemental iron affected the second phase of hypoxic pulmonary vascular response but not the first acute phase of HPV [44]. Moreover, in animal experiments long-term dietary iron restriction or genetic iron deficiency was associated with increased pulmonary vascular remodeling, enhanced pulmonary vascular resistance, and elevated PAP [283,284]. In line with these findings, in patients with chronic mountain sickness, progressive iron deficiency induced by venesection was associated with an approximately 25% increase in systolic PAP [45]. In this regard, the more common severe pulmonary hypertension in patients with chronic mountain sickness reported in earlier observations may be due to iron deficiency because of more often performed venesections in the past. Furthermore, a recent study demonstrated that alimentary iron deficiency was a possible risk factor causing right heart failure due to pulmonary hypertension in Tibetan children living in high altitude area [285].

## 7. Other Clinical Forms of Pulmonary Hypertension at High Altitude

Pulmonary hypertension can develop as a complication of a diverse range of medical conditions, including chronic lung diseases, pulmonary embolism, cardiovascular diseases, and many others. Pulmonary hypertension due to hypoxia is classified as group 3 pulmonary hypertension [286,287]. However, it should be noted that causes other than hypoxia might potentially underlie and/or contribute to pulmonary hypertension at high altitude [288].

A recent study retrospectively analyzed etiologies of pulmonary hypertension in patients admitted over five years to the Qinghai Red Cross Hospital, located at 2200 m [289]. Interestingly, in high altitude dwellers, various clinical forms of pulmonary hypertension from clinical group 1 to 5 were diagnosed [289]. The analysis showed that the most common form was pulmonary hypertension due to lung diseases [289]. Pulmonary hypertension due to chronic lung disease is also common in Andean highlanders [290]. In this regard, a recent meta-analysis found a higher prevalence of chronic obstructive pulmonary disease (COPD) at high altitudes than that from average data [291]. However, altitude itself had no significant impact on COPD prevalence [291,292]. Importantly, subjects with airflow limitation living at high altitude reported significantly fewer respiratory symptoms compared to subjects residing at lower altitude, and high altitude residence was associated with a significantly increased risk of undiagnosed COPD [292,293].

Large epidemiological studies have demonstrated high prevalence of congenital heart disease at high altitudes [294,295,296,297,298]. Birth incidence of congenital heart diseases in newborns at high altitude is about 5–40 times higher than that at low altitude [297,299,300]. The most frequent congenital heart diseases at high altitude are patent ductus arteriosus, atrial septal defect, and ventral septal defect [290,294,300]. However, in many resource-limited mountainous regions, facilities for diagnosis and treatment are sparse or unavailable [300]. Importantly, pulmonary hypertension is a relatively common complication of congenital heart disease [301,302]. Moreover, the incidence of pulmonary hypertension associated with congenital heart disease has been shown to be significantly higher at high altitude than at low altitude [290,303,304,305].

Pulmonary hypertension is a common complication of mitral valve disease and may affect more than 70% of patients depending on disease severity [306]. Mitral stenosis is a valvular heart disease that is mainly of rheumatic origin. Rheumatic heart disease is highly prevalent in many low- and middle-income countries and remains a major cause of morbidity and premature death [307,308]. Constrained access to healthcare in remote and resource-poor areas is associated with late diagnosis and referral, limited diagnostic facilities, and poor availability of appropriate therapeutic options. The prevalence rates of rheumatic heart disease are approximately tenfold higher when assessed using echocardiographic screening compared to clinical evaluation [309]. Late presentation to health facilities leads to the increased proportion of patients with advanced disease. Though earlier studies reported cases of rheumatic heart disease in high altitude dwellers [290], the prevalence of pulmonary hypertension associated with rheumatic heart disease in highlanders has not been investigated. High prevalence of the disease in endemic regions implies that a certain proportion of pulmonary hypertension cases in high altitude dwellers may be due to rheumatic heart disease.

High altitude populations are traditionally characterized by their less exposure to risk factors for coronary artery disease, such as sedentary lifestyle, smoking, high-fat diet, and stress [310]. However, with urbanization, along with broad shifts in the structure of diet and physical activity patterns of high altitude residents, cardiovascular diseases may become more prevalent. Consequently, the prevalence of pulmonary hypertension due to left heart diseases may increase among high altitude dwellers.

High altitude hypoxia is associated with an increased thrombotic risk [311]. In highlanders, chronic hypoxia-induced increases in hematocrit and erythrocytosis affect blood viscosity and impair blood flow. Moreover, elevated platelet counts, enhanced platelet adhesiveness [312,313], and shortening of clotting time [314] were reported in permanent high altitude residents. Furthermore, pulmonary hypertension due to thrombotic occlusive vascular disease was described in lowlanders after long-term acclimatization to high altitude [315]. In addition, there have been anecdotal reports of cases of chronic thromboembolic pulmonary hypertension in high altitude dwellers [288].

Thus, other common causes of secondary pulmonary hypertension in lowlanders may underlie and/or contribute to pulmonary hypertension in high altitude residents. The challenges of detection of the underlying causes of pulmonary hypertension in high altitude residents are related to the asymptomatic course of the disease, low educational level of highlanders, limited access to facilities for diagnosis, and high rates of unawareness and low index of suspicion by local physicians [293].

## 8. Prevention and Treatment of Pulmonary Hypertension in High Altitude Dwellers

Prevention of common causes of secondary pulmonary hypertension can prevent development of pulmonary hypertension in high altitude dwellers. Early diagnosis and timely treatment of a bacterial sore throat can help prevent rheumatic fever and subsequently pulmonary hypertension due to rheumatic heart disease. Implementing preventive screening programs for congenital heart disease and rheumatic heart disease using echocardiography can facilitate early detection and treatment of these diseases.

The goal of the management of pulmonary hypertension is primary therapy of the underlying cause. Before treatment initiation, all efforts should be made to diagnose the underlying condition that may be responsible for pulmonary hypertension in a given case. Therefore, the awareness of local physicians of other clinical forms of pulmonary hypertension is very important in order to consider all the potential causes of pulmonary hypertension in every patient using the classic diagnostic tools, including careful clinical history taking and physical examination complemented by laboratory and imaging methods.

Given the important role of hypoxia in the pathogenesis of hypoxic pulmonary hypertension, relocation of the place of residence to lower altitudes may improve pulmonary hypertension and probably cure the disease. Earlier studies have demonstrated a reduction of PAP in healthy highlanders after two years of relocation to low altitude [316,317]. Likewise, in most temporary high altitude residents, symptoms of pulmonary hypertension gradually resolve upon return to sea level [174].

### 8.1. Potential of Drugs Approved for Treatment of Pulmonary Arterial Hypertension

Descent to low altitudes may not be acceptable to high altitude dwellers for various reasons, necessitating administration of pharmacological therapies. However, no drugs have been approved to date for the therapy of chronic high altitude pulmonary hypertension. Extensive clinical experience with vasodilator drugs in patients with pulmonary arterial hypertension suggests their potential for the treatment of high altitude pulmonary hypertension. However, only few studies have investigated the effects of drug therapy on pulmonary hypertension in high altitude dwellers. Small studies have demonstrated efficacy of single doses of nifedipine [198] and bosentan [318] in reducing PAP in high altitude dwellers with mild-to-moderate PAP elevation. Further, a randomized double-blind placebo-controlled trial demonstrated that 12-week treatment with sildenafil in high altitude dwellers was associated with a significant reduction in PAP and increased cardiac output and 6 min walk distance [319].

### 8.2. Drug Repurposing

Since most high altitude populations live in resource-limited countries, access to the currently approved pulmonary arterial hypertension pharmacologic therapies may be limited by their availability and their cost [320,321]. Therefore, discovery of inexpensive treatment strategies by repurposing drugs that are already used for other medical conditions might represent another attractive option [322,323].

Acetazolamide is a carbonic anhydrase inhibitor that is used in the prevention of acute mountain sickness. Experimental studies demonstrated that acetazolamide prevented hematocrit elevation and pulmonary vascular resistance increase in chronically hypoxic rats [324]. Importantly, administration of acetazolamide in rats with established hypoxic pulmonary hypertension was also able to reduce hematocrit and pulmonary vascular resistance [324]. In patients with chronic mountain sickness, long-term acetazolamide administration in a dose of 250 mg/day resulted in cardiac output increase and reduction in pulmonary vascular resistance [272]. Given its wide availability, low cost, and favorable side effect profile, acetazolamide has the potential for therapy of pulmonary hypertension in highlanders with chronic mountain sickness.

Compelling experimental evidence suggests an important role Rho-kinase in the pathogenesis of pulmonary hypertension [325]. Fasudil is a potent Rho-kinase inhibitor and vasodilator that has been approved for the treatment of cerebral vasospasm [326]. In various animal models of hypoxic pulmonary hypertension, fasudil reduced PAP and pulmonary vascular resistance [327,328,329,330,331]. Furthermore, the short-term efficacy and safety of fasudil in the treatment of pulmonary arterial hypertension patients were demonstrated in clinical trials [332,333,334,335]. A small study has demonstrated efficacy of a single dose of fasudil in reducing PAP in high altitude dwellers with mild-to-moderate PAP elevation [336].

### 8.3. Novel Potential Therapeutic Targets

Experimental studies using hypoxia-induced pulmonary hypertension models help in identifying new targets for development of novel drugs, including growth factors, transcription factors, and inflammation and metabolic remodeling [337].

Among numerous growth factors, an important role in the pathogenesis of hypoxic pulmonary hypertension has been demonstrated for the platelet-derived growth factor [338,339,340], bone morphogenetic protein type 2 receptor [341,342,343,344,345], fibroblast growth factor [346,347], transforming growth factor [341,348,349,350], vascular endothelial growth factor [346,351,352,353] angiopoietin [354,355].

Hypoxia activates various transcription factors and the hypoxia-inducible factor is the most prominent among them [223,356]. Other transcription factors involved in the pathogenesis of hypoxic pulmonary hypertension include Notch [357,358], Fox [359,360] peroxisome proliferator-activated receptors [361,362,363,364].

Accumulating evidence suggests a role of inflammation and oxidative stress in hypoxic pulmonary hypertension [365,366,367,368]. Recent experimental studies have demonstrated efficacy of direct anti-inflammatory and oxidative stress reducing drugs in attenuating pulmonary hypertension. Clinical trials evaluating these drugs in pulmonary hypertension patients are ongoing [369]. There is also substantial evidence implicating abnormal metabolism in the pathogenesis of pulmonary hypertension [370]. Consequently, emerging therapeutic interventions targeting aberrant metabolic pathways have shown a great potential of this approach in preclinical models of hypoxic pulmonary hypertension [371].

## 9. Summary

Hypoxic pulmonary hypertension plays an important role in the pathogenesis of HAPE. Drugs that increase nitric oxide bioavailability or attenuate endothelin-1 signaling have shown potential to blunt PAP increase in response to acute hypoxia or at high altitude in healthy individuals. Currently, due to extensive clinical experience with nifedipine in HAPE prevention in susceptible individuals, only calcium-channel blockers are recommended for drug prophylaxis in high-risk individuals with a clear history of recurrent HAPE. Further controlled studies are necessary to evaluate the efficacy of the vasodilator therapy in HAPE treatment in susceptible individuals.

While in most altitude dwellers elevation of PAP is mild-to-moderate, some of the highlanders develop pulmonary hypertension. Pulmonary vascular disease in high altitude residents may be due to the effects of hypobaric hypoxia and/or may have other underlying causes, such as congenital heart disease, rheumatic heart disease, chronic lung diseases, left heart diseases, and others. Several small studies have investigated the potential of drug therapy of pulmonary hypertension in high altitude dwellers and have had positive results. However, no drugs have been approved for the therapy of chronic high altitude pulmonary hypertension yet. Preclinical studies provide new insights into the role of various signaling pathways involved in the pathogenesis of hypoxic pulmonary hypertension, which may serve as promising therapeutic targets.

## Figures and Tables

**Table 1:** Summary of relevant studies evaluating effects of potential drugs on HPV in in vitro, ex vivo, and in vivo rodent models.

Drugs	Mechanism of Action	Effects	References
Inhaled NO	Soluble guanylate cyclase stimulator	attenuates hypoxia-induced PVR increase in ventilated dogs	[139]
		improves survival of HAPE rats at extreme altitude (hypobaric chamber)	[140]
		decreases PAP and PVR in hypoxia-breathing lambs	[105,141]
Sildenafil	Phosphodiesterase 5 inhibitor	attenuates hypoxia-induced human and rats PA constriction	[142]
		attenuates hypoxia-induced RVSP increase in ventilated rats	[143]
Tadalafil	Phosphodiesterase 5 inhibitor	inhibits hypoxia-induced isolated rat PA constriction	[144]
		prevents an increase in RVSP in ventilated hypoxia-breathing rats	[145]
Bosentan	Endothelin receptor receptor A/B antagonist	decreases PAP in isolated perfused rat lung	[146]
		prevents PAP increase in hypoxia exposed rats	[147]
Nifedipine	Calcium channel blocker	reduces PVR in hypoxia-breathing awake piglets	[148]
Dexamethasone	Anti-inflammatory and immunosuppressive agent	increases NOS expression and improves hypoxia-induced PAEC dysfunction	[130]
		stimulates the expression of Na transporters and prevents hypoxia-induced inhibition of alveolar reabsorption	[133]
		improves lung epithelial Na^+^ channels and Na^+^/K^+^-ATPase and increases alveolar fluid clearance in rats	[132]
		improves pulmonary capillary endothelial permeability and prevents HAPE development in rat model	[131]
Acetazolamide	Carbonic anhydrase inhibitor	prevents an increase in PAP and PVR in conscious hypoxia-breathing dogs	[149]
		inhibits hypoxia-induced Ca^2+^ influx in PASMCs	[150]

PA, pulmonary artery; PAP, pulmonary artery pressure; RVSP, right ventricular systolic pressure; PVR, pulmonary vascular resistance; HAPE, high altitude pulmonary edema; NOS, nitric oxide synthase; PAEC, pulmonary artery endothelial cell; PASMC, pulmonary artery smooth muscle cell.

**Table 2:** Summary of relevant studies evaluating effects of potential drugs on HPV in healthy human subjects exposed to hypoxia.

Drugs	Mechanism of Action	Effects	References
Inhaled NO	Soluble guanylate cyclase stimulator	decreases sPAP and improves oxygenation in HAPE-prone subjects at HA	[112]
		reduces mPAP and PVR in HAPE patients at HA	[113]
		decreases PAP and PVR in hypoxia-breathing healthy subjects	[112]
Sildenafil	Phosphodiesterase 5 inhibitor	prevents an increase in PAP and PVR in hypoxia-breathing healthy subjects	[20,151]
		decreases sPAP and improves exercise capacity under hypoxic breathing and at HA in healthy subjects	[114]
		decreases sPAP and improves blood oxygenation upon acute HA exposure in healthy subjects	[117]
		attenuates sPAP increase and RV dysfunction upon acute HA exposure in healthy subjects	[22]
Tadalafil	Phosphodiesterase 5 inhibitor	attenuates an increase in PAP and prevents HAPE development	[64]
Bosentan	Endothelin receptor receptor A/B antagonist	prevents an increase in sPAP and PVR in hypoxia breathing healthy subjects	[125,126,127]
		prevents an increase in sPAP and improves arterial oxygen saturation in healthy subjects exposed to HA	[122]
Sitaxentan	Endothelin receptor antagonist	prevents an increase of PAP and PVR and exercise capacity decline in hypoxia-breathing healthy subjects	[129]
		reduces PVR and improves lung diffusion capacity and exercise capacity in healthy subjects exposed to HA	[152]
		reduces PAP and PVR and improves exercise capacity in healthy subjects exposed to HA	[129]
Nifedipine	Calcium channel blocker	reduces PAP and improves HAPE symptoms	[66]
		attenuates an increase in PAP and prevents HAPE development	[63]
Dexamethasone	Anti-inflammatory and immunosuppressive agent	attenuates an increase in PAP and prevents HAPE development	[64]
		attenuates an increase in TRG and PVR and improves oxygenation in COPD patients at HA	[153]
		attenuates an increase in TRG and improves exercise capacity	[154]
Acetazolamide	Carbonic anhydrase inhibitor	prevents an increase in TRG in hypoxia-breathing healthy subjects	[136]

HA, high altitude; PAP, pulmonary artery pressure; sPAP, systolic pulmonary artery pressure; RVSP, right ventricular systolic pressure; TRG, tricuspid regurgitation gradient; PVR, pulmonary vascular resistance; HAPE, high altitude pulmonary edema; COPD, chronic obstructive pulmonary disease; NOS, nitric oxide synthase; PAEC, pulmonary artery endothelial cell; PASMC, pulmonary artery smooth muscle cell.

## Data Availability

Not applicable.
